# Measuring within-day cognitive performance using the experience sampling method: A pilot study in a healthy population

**DOI:** 10.1371/journal.pone.0226409

**Published:** 2019-12-12

**Authors:** Simone J. W. Verhagen, Naomi E. M. Daniëls, Sara Laureen Bartels, Sulina Tans, Karel W. H. Borkelmans, Marjolein E. de Vugt, Philippe A. E. G. Delespaul

**Affiliations:** 1 Department of Psychiatry and Neuropsychology, Faculty of Health, Medicine and Life Sciences, Maastricht University, Maastricht, the Netherlands; 2 Department of Family Medicine, Faculty of Health, Medicine and Life Sciences, Maastricht University, Maastricht, The Netherlands; 3 Alzheimer Centre Limburg, School for Mental Health and Neuroscience, Maastricht University, Maastricht, the Netherlands; 4 Mondriaan Mental Health Trust, Department of Adult Psychiatry, Heerlen, the Netherlands; Lluita contra la SIDA Foundation - Germans Trias i Pujol University Hospital - Autònoma de Barcelona University, SPAIN

## Abstract

**Introduction:**

People with depression, anxiety, or psychosis often complain of confusion, problems concentrating or difficulties cognitively appraising contextual cues. The same applies to people with neurodegenerative diseases or brain damage such as dementia or stroke. Assessments of those cognitive difficulties often occurs in cross-sectional and controlled clinical settings. Information on daily moment-to-moment cognitive fluctuations and its relation to affect and context is lacking. The development and evaluation of a digital cognition task is presented. It enables the fine-grained mapping of cognition and its relation to mood, intrapersonal factors and context.

**Methods:**

The momentary Digit Symbol Substitution Task is a modified digital version of the original paper-and-pencil task, with a duration of 30 seconds and implemented in an experience sampling protocol (8 semi-random assessments a day on 6 consecutive days). It was tested in the healthy population (*N* = 40). Descriptive statistics and multilevel regression analyses were used to determine initial feasibility and assess cognitive patterns in everyday life. Cognition outcome measures were the number of trials within the 30-second sessions and the percentage of correct trials.

**Results:**

Subjects reported the task to be easy, pleasant and do-able. On average, participants completed 11 trials with 97% accuracy per 30-second session. Cognitive variation was related to mood, with an interaction between positive and negative affect for accuracy (% correct) (*p* = .001) and an association between positive affect and speed (number of trials) (*p* = .01). Specifically, cheerful, irritated and anxious seem to covary with cognition. Distraction and location are relevant contextual factors. The number of trials showed a learning effect (*p* < .001) and was sensitive to age (*p* < .001).

**Conclusion:**

Implementing a digital cognition task within an experience-sampling paradigm shows promise. Fine-tuning in further research and in clinical samples is needed. Gaining insight into cognitive functioning could help patients navigate and adjust the demands of daily life.

## Introduction

Various patient populations experience confusion, difficulties to concentrate or problems to cognitively grasp contextual cues[1–5]. To assess an individual’s ability to function and cope in everyday life, neuropsychological tests are crucial. The information generated can be included in evaluating whether someone is, for example, capable of independent living or self-care[6]. Given these far-reaching consequences, it is important that the performance measured with a neuropsychological test accurately reflects performance in daily life. A review by Chaytor and Schmitter-Edgecombe [2003] suggests, however, that, when the relationship between tests and measures of daily functioning is considered, neuropsychological tests might only have moderate ecological validity for predicting everyday cognitive functioning[7].

While the general use of neuropsychological tests has gained importance in recent years, the tests themselves as well as the standardized context of administration remained largely the same[8]. Often, a battery of cognition tests (e.g., CANTAB) are used to determine someone’s cognitive potential on a range of domains[9]. Individual tests often take several minutes to administer and are performed in the presence of a professional in minimum distraction environments. The goal is to determine a stable cognition factor that provides insight into the individual’s general strengths and vulnerabilities[8]. However, the clinical test conditions sharply contrast with everyday environments. Everyday life is comprised of multi-sensory elements such as distracting sounds, smells, lights, or tactile stimuli. Furthermore, daily stressors and mental states can influence an individual’s cognitive ability[10, 11]. Mood, for example, follows a dynamic pattern in everyday life[12] and its effect on cognition from one moment to the next is seldom considered. Moreover, cognition is known to fluctuate over the day, depending on factors such as the level of alertness or food intake[13, 14]. To improve the understanding of cognition in everyday life, the assessments need to take place in natural daily environments. Ideally, other domains such as mood and behavior are monitored simultaneously so that underlying associations can be learned. Insight into these implicit patterns would enrich treatment for cognitive complaints and provide additional clues for recovery and rehabilitation processes, next to opportunities to tailor interventions to the individual[15]. By providing cognitive assessments within the Experience Sampling Method (ESM) this strategy becomes possible.

ESM, also called Ecological Momentary Assessments (EMA), is a (digital) structured self-assessment diary technique that allows insight into the everyday life of an individual[16]. At several (semi-) random times during the day, eHealth technologies such as Personal Digital Assistants or smartphone apps give signals (beeps) to prompt the collection of momentary experiences. At those moments, participants are asked to reflect on their current mood, environmental context, and activities and report their real-time information to the eHealth technology used. ESM is characterized by a high ecological validity as it collects experiential and contextual data in situ[17]. In-the-moment reflections reduce the recall bias that troubles retrospective self-reports[18]. Furthermore, repeated ESM measures allow a better understanding of between- and within-person variability in psychopathology and beyond[12]. As ESM can be experienced as time-consuming, the questionnaires need to be kept short and the design transparent to avoid overburdening[17].

The initial feasibility and acceptability of cognition tasks in an ESM paradigm are supported by a small number of studies, including domains such as working memory, attention, or processing speed[19]. The feasibility of a digital trail making test assessing processing speed in everyday life, for example, was found to be feasible in Chinese patients with depression[20]. Another study investigated the reliability and validity of three ambulatory cognition tasks measuring different cognitive domains (i.e., Symbol search, Dot memory, and an N-back task)[21]. Results indicated that all three tasks are feasible within an ESM paradigm and show excellent between-person reliability, reliable within-person variability, and construct validity with cross-sectional cognitive assessments[21]. In young adults, a digital processing speed task was not only feasible, but also sensitive to blood alcohol concentration[22].

Notably, most studies on daily life cognition focus only on a limited number of contextual factors in relation to cognitive performance. As everyday life is extremely complex, more research is needed to contextualize daily cognition with extensive intrapersonal (e.g., mood, age, fatigue) and contextual factors (e.g., location, company). Additionally, cognition tasks in everyday environments that take multiple minutes to perform[23] might, on one hand, provide valuable information on daily cognitive functioning. On the other hand, the length of the task can result in a relatively low sampling frequency to not overburden the participant and thus limit the exploration of cognitive fluctuations over the course of the day. In order to learn which factors influence cognitive variation over time, a higher sampling rate is required with shorter beep durations to minimize burden. This strategy would enable to study the influence of different daily situations on cognition. Ultimately, the test results could be reported back to patients and discussed together with a clinician in relation to other relevant health domains.

The present study aims to build an objective cognition task with a short duration for repeated assessments and to implement this task into a daily life setting. Accordingly, a modified digital version of the Digital Symbol Substitution Task was used within the ESM-based PsyMate^™^ application on an iPod for six consecutive days by healthy individuals. This digital cognition task is called momentary Digital Symbol Sustitition Task (mDSST).

First, the utility and feasibility of the mDSST was determined through the participants’ compliance rate and retrospective subjective experience. Second, the focus lay on validation via comprehensive contextualization of daily cognitive performance. The relationship between intrapersonal as well as contextual factors and the mDSST performance was investigated using high frequency ESM sampling (eight times a day).

Prospectively, digital cognition tasks in everyday life may be relevant for improved prevention, treatment, and rehabilitation of psychopathology.

## Methods

### Participants

Individuals from the general population were recruited via poster advertisement at Maastricht University and through social media as seeds for snowball sampling[24]. Sample size was based on recommendations for pilot studies and other exploratory ESM studies[25–27]. In total, 45 participants provided written informed consent. All individuals were 18 years or older, had sufficient command of the Dutch language, and were able to handle an iPod with the PsyMate^™^ app. Exclusion criteria were medication use that influences cognitive performance and current treatment for mental illnesses or cognitive complaints. Ethical approval was obtained by the standing ethical committee of the Faculty of Psychology and Neuroscience, Maastricht University (ref.no.183_02_09_2017).

### Measurements

#### PsyMate^™^

The PsyMate^™^ is a web-based platform for moment-to-moment assessment of mood and behavior in daily life. It includes an App (iOS and Android), cloud-based data storage, and reporting tool. The PsyMate^™^ was developed by Maastricht University and Maastricht UMC+ (www.psymate.eu) and programmed to prompt participants using auditory signals eight times a day to complete a self-report questionnaire (approximately two minutes). Signals (beeps) were provided between 7.30 AM and 10.30 PM in semi-random time blocks of 112,5 minutes. The self-report questionnaire assessed mood, physical status (i.e., fatigue, hunger), and context (i.e., location, activity, and persons present). The mood items were combined in two independent constructs[15]: Positive Affect (PA) by averaging ‘cheerful’, ‘energetic’, ‘relaxed’, ‘enthusiastic’, and ‘satisfied’, and Negative Affect (NA) using ‘insecure’, ‘down’, ‘irritated’, ‘lonely’, ‘anxious’, and ‘guilty’. The mood and physical status items were rated on a 7-point Likert scale (1 = not at all, 4 = moderate, 7 = very) and the context items were assessed categorically. The complete item list is included as supporting information (see S1 Appendix). In addition to the self-report questionnaire on the beep level, participants were asked to complete a morning and an evening questionnaire. These additional questionnaires consisted of self-report items that assessed respectively sleep duration and sleep quality, and general appraisal of the day. Most items of the morning questionnaire were assessed categorically, whereas all the items of the evening questionnaire were rated on a 7-point Likert scale (1 = not at all, 4 = moderate, 7 = very). Participants were included in the analyses if they completed a minimum of sixteen valid beep moments (1/3 of total), conform with ESM guidelines[28]. All participants were provided with an iPod on which the PsyMate^™^ app (version 2.0.0.) was installed to standardize the administration of the momentary Digit Symbol Substitution Task (mDSST). To evaluate the PsyMate^™^ procedure, debriefing questionnaires were provided after the ESM completion.

#### PsyMate^™^ mDSST

The mDSST is based on the Digit Symbol Substitution Task from the Wechsler Adult Intelligence Scale (WAIS)[29]. It measures information processing speed and short-term working memory. The modified mDSST primarily assesses information processing speed, but not short-term working memory due to design choices (e.g., short duration, one-by-one presentation) that are part of the ESM set-up. The task was selected after consultations with psychiatric and neuropsychological healthcare professionals and scholars of daily life assessment. The constraints were that the digital cognition task could be performed multiple times per day and therefore had to be short, sensitive to cognitive fluctuations, and show no or only a small learning effect. The mDSST is thought to fulfill these criteria.

**Fig 1:**
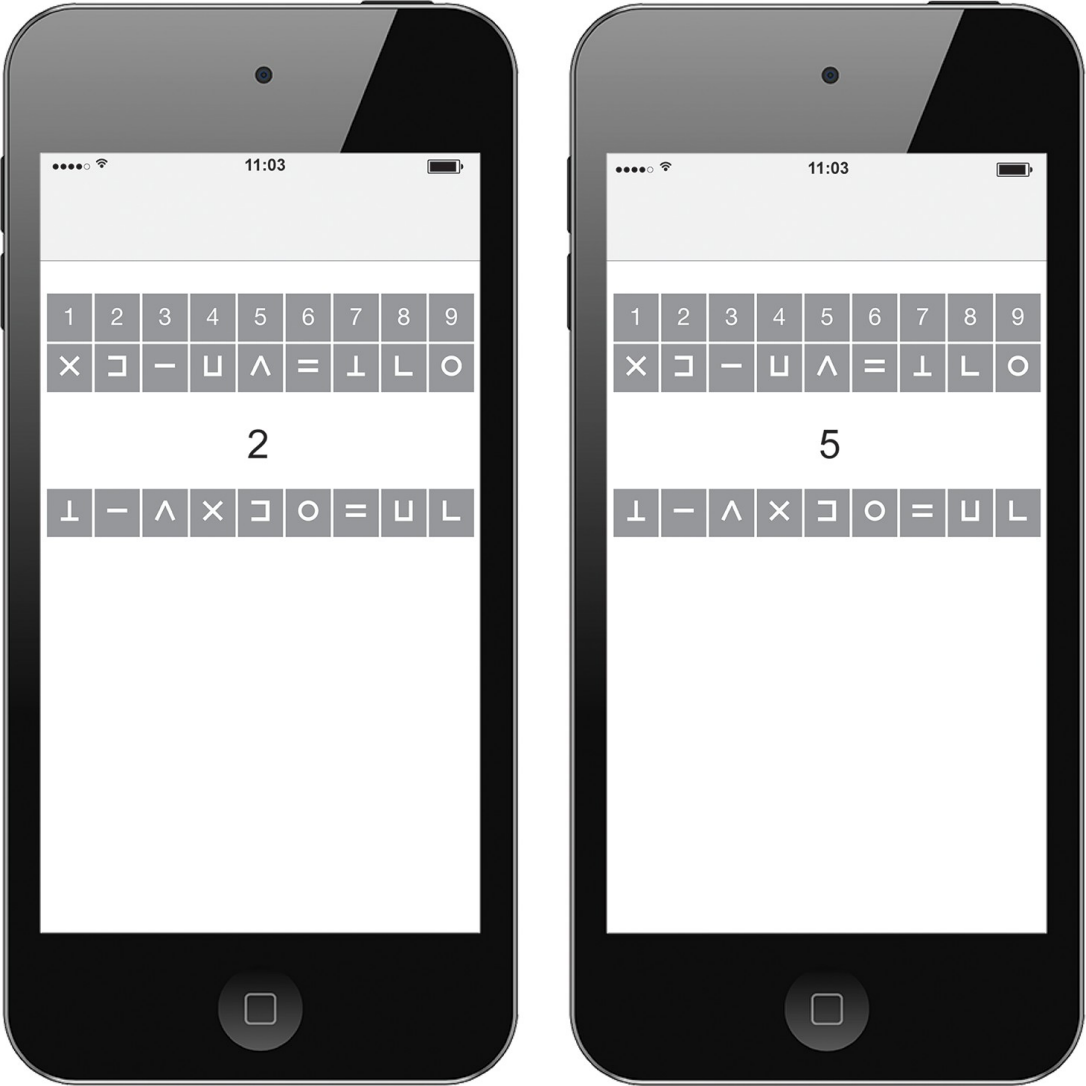
Momentary digit symbol substitution task in the PsyMate^™^ application.

The mDSST started after the standard ESM beep questionnaire. Participants viewed an instruction screen including a button to start the task. The item screen displays the numbers 1 to 9 with a corresponding symbol at the top of the screen (encoding information). For each trial, a number was presented one-by-one in the middle of the screen. Participants had to select the corresponding symbol at the bottom of the screen (see Fig 1). Symbols were kept similar to the original Digit Symbol Substitution Task. The task duration was 30 seconds and participants were instructed to complete as many trials as possible while also being as accurate as possible. Five unique combinations of numbers and symbols with corresponding answer keys were programmed beforehand and presented in random order over the course of the 48 beeps. Outcome measures of the PsyMate^™^ mDSST are the number of trials (how many one-by-one trials are completed within 30-second sessions) and the percentage of correct trials (the number of correctly answered trials divided by the total number of trials).

#### Debriefing questionnaire

Participants received a debriefing questionnaire with three parts using open-ended and 7-point Likert scale questions: 1) to assess the general experience of participants throughout the week (e.g., was this a normal week, did participation influence your mood, social contact or activities); 2) to evaluate the usability of the PsyMate^™^ in general (e.g., was the PsyMate^™^ difficult to use, was the number of questionnaires burdensome, were there any technical issues); and 3) to assess the experiences with the mDSST (e.g., how well do you think you performed on the task, was the task difficult, was the task enjoyable).

### Procedure

After participants provided written informed consent, a briefing session of one hour took place. Participants provided sociodemographic information including gender, age, living situation, education level, current occupation, and ethnicity. Additionally, current medication use and treatment for mental illnesses and cognitive complaints were assessed through self-reports. Furthermore, participants received an iPod (5^th^ generation) with the PsyMate^™^ (v2.0.0.) preinstalled. They were instructed how to use the PsyMate^™^ and performed a test trial to familiarize themselves with the ESM procedure. Then, the participants used the PsyMate^™^ for six consecutive days, starting on the day after the briefing session. On the second day of the ESM period, participants were contacted by telephone to assist with potential problems or answer questions. After the ESM period, a debriefing session of one hour took place in which participants completed the debriefing questionnaire and returned the iPod.

### Statistical analyses

Descriptive statistics were used to assess participant characteristics, initial feasibility, and acceptability (frequencies) of the ESM protocol. The completion rate was calculated by comparing the mean percentage of valid beep moments to the total number of beep moments. The data collected with the PsyMate^™^ have a multilevel structure; beeps (level 1) were nested in participants (level 2). Average scores of the variables of interest were person-mean centered to take into account the within-person effect. In order to look at contextualized variation, dummy variables were created for location (at home versus somewhere else), company (alone versus with others), and coffee use since the last beep (yes or no). Furthermore, activity-related stress was conceptualized as an average of the items ‘I would rather be doing something else’, ‘This is difficult for me’, and ‘I can do this well’ (reverse coded). In order to look at learning effects, a log transformation of the replication (sequence number of responded beeps within subjects ranging from 1 (first beep) to 48 (last beep)) was calculated as a proxy measure of time across the six day period. Additionally, within-day time effect was explored using hour of the day and its quadratic function. To assess cognitive variation over time and to check for learning effects, multilevel regression analyses were run with the number of trials within the 30-seconds interval and the percentage of correct trials (for each assessment moment) on the mDSST as dependent variables and respectively time (i.e., log transformation of replication), hour, squared hour, and a log transformation of day number (from day 1 to day 6) as independent variables. Furthermore, multilevel analyses were run to assess the association between positive affect, negative affect, its interaction, and various other contextual factors (e.g., fatigue, distraction) as independent variables and both cognition outcomes as dependent variables. Additionally, multilevel stepwise regression procedures were used to explore the effect of individual mood items on cognition. Both forward and backward strategies were applied. The individual mood items and various other contextual factors were seen as independent variables and cognition as dependent variable. Quadratic function of age, gender, possible learning effects, and within-day effects were considered as covariates in all multilevel models. Analyses were carried out using Stata version 13.0[30]. A two-sided significance level of .05 was used.

## Results

### Participants

Forty-five participants were included in the ESM protocol, resulting in 1330 valid beep records. Two participants were unable to finish the ESM protocol due to problems with the iPod device (loss of 11 records, 0.83%), one participant was excluded because the iPod was stolen (loss of 9 records, 0.68%), and two participants did not reach the criteria of at least 16 valid beeps due to various reasons (loss of 20 records, 1.50%). The complete dataset consisted of 40 participants with 1293 valid beep records. The participants’ age ranged from 21 to 72 years of age with a mean of 30.4 (*SD* = 14.79, *Mdn* = 23.0). On average, participants completed 33 beeps (*SD* = 4.9, range 21–43) of the 48 scheduled beeps. ESM completion rate was 69%. See Table 1 for descriptive statistics of the healthy population sample.

**Table 1:** Descriptive statistics for the healthy population sample (N = 40).

	N (%)
**Gender (women)**	29 (72.5%)
**Education level**	
** Secondary vocational education**	5 (13%)
** Bachelor degree**	20 (50%)
** Master degree**	15 (37%)
**Occupation**	
** Students**	25 (62%)
** Fulltime work**	10 (25%)
** Part-time work**	3 (8%)
** Voluntary work**	1 (2,5%)
** No occupation**	1 (2,5%)

### Feasibility

In order to assess feasibility, the available data from the debriefing questionnaire was used. One participant, whose iPod was stolen, did not complete this evaluation questionnaire, leaving 44 participants in the sample. All other analyses based on ESM/PsyMate^™^data were performed with a sample size of forty participants.

#### Evaluation PsyMate^™^ procedure

Participants reported that the ESM items were a good representation of their experience (*M* = 5.1, *SD* = 1.26). They had no difficulty using the PsyMate^™^ (*M* = 1.59, *SD* = 1.06) and the verbal and written instructions were clear (verbal: *M* = 6.64, *SD* = .53; written: *M* = 6.43, *SD* = .70). Furthermore, completing the items had little influence on their mood (*M* = 2.07, *SD* = 1.26), activities (*M* = 1.89, *SD* = 1.5), and social contact (*M* = 1.55, *SD* = .93). Participating in ESM did not hinder their daily activities (*M* = 2.16, *SD* = 1.31). With regard to the burden, participants reported that the number of beeps a day (*M* = 3.23, *SD* = 1.46), the duration of beep completion (*M* = 2.32, *SD* = 1.29), and the beep sound (*M* = 3.18, *SD* = 1.97) had low impact.

#### Evaluation of the mDSST

Participants were motivated to perform well on the mDSST (*M* = 5.70, *SD* = .93), the mDSST was moderately pleasant to perform (*M* = 4.43, *SD* = 1.37), and participants would recommend the task to others (*M* = 5.48, *SD* = 1.17). Overall, the task was experienced as easy (*M* = 1.80, *SD* = 1.15). However, when participants had to assess their own performance retrospectively, they indicated to have performed moderately on the mDSST (*M* = 4.55, *SD* = 1.19). Also in retrospect, they reported to be moderately distracted during the task (*M* = 3.51, *SD* = 1.39).

### Variation in cognition

Participants completed on average 11.39 trials within 30-second sessions (*SD* = 1.32,range 3–15), with an average percentage correct of 97.11 (*SD* = 2.01, range 28.6–100). The number of trials was positively associated with time (*B* = .36, *SE* = .033, *p* < .001, 95% CI = .30, .43), with a positive within-day effect for hour of the day (*B* = .03, *SE* = .007, *p* < .001, 95% CI = .01, .04), and a positive between-day effect for day number (*B* = .45, *SE* = .05, *p* < .001, 95% CI = .35, .54). The percentage of correct trials was not associated with time (*B* = -.26, *SE* = .19, *p* = .17, 95% CI = -.63, .11), with no within-day (*B* = -.04, *SE* = .04, *p* = .36, 95% CI = -.11, .04) or between-day effect (*B* = -.21, *SE* = .27, *p* = .45, 95% CI = -.74, .32).

### Mood, contextual factors, and cognition

Participants experienced high positive affect (*M* = 4.82, *SD* = .77, range 2.68–6.48) and low negative affect (*M* = 1.65, *SD* = .47, range 1.01–2.98) throughout the study. They were a little worried (*M* = 2.52, *SD* = 1.00, range 1.00–4.78) and felt moderately fatigued (*M* = 3.69, *SD* = 1.03, range 1.55–5.77). Furthermore, they experienced low activity-related stress (*M* = 2.68, *SD* = .61, range 1.37–3.73) and were moderately focused on their current activities (*M* = 4.87, *SD* = .73, range 3.39–6.63). On the mDSST, they reported a low to moderate level of distraction during this task (*M* = 2.88, *SD* = .88, range 1.15–4.67).

Only the main significant aggregated findings from the multilevel regression analyses are reported. Single-item analyses are included in the supplementary material (see S1 Table). Participants performed more trials (*B* = .08, *p* = .04) and made less mistakes (*B* = .62, *p* = .001) when experiencing high positive affect. They made more mistakes when experiencing high negative affect (*B* = -1.41, *p* < .001).With regard to the contextual factors, participants performed less trials when being at a different location then home (*B* = -.20, *p* = .002) and when reporting to be distracted (*B* = -.17, *p* < .001). They also made more mistakes when distracted (*B* = -.46, *p* < .001). Fatigue, activity-related stress, worrying, current company, coffee use, and being able to focus were unrelated to both cognition outcome measures. With regard to possible covariates, less trials were performed with higher age (*B* = -.001, *p* < .001), and when being male. A positive association was found between the time measures (i.e., the log-transformed replication variable as time measure, hour, squared hour) and the number of trials (e.g., the log-transformed replication variable as time measure; *B* = .36, *p* < .001). The variables with an association with the cognitive outcome measures were included in further multilevel regression models.

In the final model of the number of trials, participants again performed more trials when experiencing high positive affect (*B* = .20, *p* = .01). In addition, a positive learing effect was present with more trials completed over time (*B* = .38, *p* < .001). Moreover, participants completed less trials when distracted (*B* = -.19, *p* < .001) and at an older age (*B* = -.0008, *p* < .001). The results of this analysis indicated that the six predictors explained 36% of the overall variance (16% within-subject variance and 47% between-subject variance).

In the final model of the percentage of correct trials, a positive interaction effect was found between positive affect and negative affect for the percentage of correct trials. In other words, the influence of negative affect on correctness is limited when positive affect is high, but stronger when positive affect is low (*B* = .71, *p* = .001). Additionally, participants made more mistakes when distracted (*B* = -.46, *p* < .001). The results of this analysis indicated that the four predictors explained 3% of the overall variance (5% within-subject variance and 0.1% between-subject variance). The results of the final models are presented in Table 2 (the number of trials) and Table 3 (the percentage of correct trials).

**Table 2:** Multilevel regression analyses of mood, distraction, time, and age during the mDSST on the number of trials.

	Number of trials				
	B	SE	p	95% CI	
**Model 1**			< .001*		
**Positive Affect**	.20	.08	.01*	.04,	.36
**Negative Affect**	.27	.17	.12	-.07,	.60
**Interaction between Positive Affect and Negative Affect**	-.04	.04	.34	-.12,	.04
**Distracted**	-.19	.02	< .001*	-.22,	-.15
**Time**^**$**^	.38	.03	< .001*	.31,	.44
**Age**^**2**^	-.0008	.0001	< .001*	-.001,	-.0005

*Note*. CI = Confidence Interval

Time^$^ = log-transformed replication score

Age^2^ = squared age.

*p < .05.

**Table 3:** Multilevel regression analyses of mood and distraction during the mDSST on the percentage of correct trials.

	Percentage of correct trials				
	B	SE	p	95% CI	
**Model 1**			< .001*		
**Positive Affect**	-.89	.43	.04*	-1.73,	-.05
**Negative Affect**	-4.10	.97	< .001*	-5.99,	-2.21
**Interaction between Positive Affect and Negative Affect**	.71	.22	.001*	.28,	1.15
**Distracted**	-.46	.11	< .001*	-.67,	-.26

*Note*. CI = Confidence Interval.

*p < .05.

### Exploratory analyses on individual mood items

The pairwise correlation of individual mood items ranged from .42 to .74 for positive affect items and from .30 to .54 for negative affect items. These correlations disregard the nested within-subject variance. When substracting by subject means to assess within-subject variance only, the correlations were considerably lower (from .24 to .63 for positive affect, and from .18 to .40 for negative affect). Results are presented in the supporting information (see S2 Table).

Exploratory multilevel regression analyses of individual mood items on cognition were computed, using mood items as independent variables and cognitive outcome measures as dependent variables (see S3 Table for an overview). Only the items cheerful and energetic were positively associated with the number of trials (respectively *B* = .12, *p* < .001; *B* = .06, *p* = .02). The positive affect items cheerful (*B* = .54, *p* < .001), relaxed (*B* = .51, *p* < .001), and satisfied (*B* = .53, *p* = .001) were positively associated with the percentage of correct trials. All negative affect items were negatively associated with percentage of correct trials.

In order to weigh item covariation, both forward and backward stepwise strategies were applied. These results are also presented in the supporting information (see S3 Table). In the backward-approach, cheerful remained the most prominent positive mood variable associated with the number of trials (*B* = .13 *p* < .001) and the percentage of correct trials (*B* = .36 *p* = .03). For the negative affect items, irritated showed a positive association with the number of trials (*B* = .07 *p* = .01), whereas anxious was negatively associated with the percentage of correct trials (*B* = -.69 *p* = .01).

## Discussion

A novel digital cognition task, the mDSST, was evaluated for use within a daily life ESM protocol. The first aim was to assess the utility and initial feasibility of the mDSST. The second aim was to study the preliminary internal validation of measuring cognition in daily life, both as varying over time and in relation to contextual and intrapersonal factors.

### Feasibility and utility of the PsyMate^™^ mDSST

ESM data from three participants were removed due to circumstances outside our control and two participants did not reach the minimum beep requirements, leaving 40 participants with analyzable data. Participants completed on average 33 beeps within a 48-beep protocol, resulting in a completion rate of 69%. The participants’ overall experience was positive; ESM completion did not hinder daily life and the burden was reported as acceptable. This result is satisfactory and similar to other ESM research with and without a cognition task[19, 23, 31, 32]. The cognition task was evaluated as easy and pleasant to perform. Task motivation was high and participants felt competitive towards the task, although several participants indicated that this competitiveness faded towards the end of the six day assessment period. This is an indication that the task is less suited for longer datacollection periods, as is relevant in clinical practice. Solutions in this context should alternate the task with another cognition measure or provide cognitive assessments in a subset of beep-moments each day.

### Contextualization of the PsyMate mDSST

Information processing speed was measured with a modified momentary version of the Digit Symbol Subtitution Task that yielded two outcome measures: the number of trials within 30 seconds and the percentage of correct trials[29]. On average, participants completed 11 trials within 30-second sessions (speed) and answered 97% correct (accuracy). This high correctness score indicates that the task is easy, something that is also reflected in the participants’ retrospective evaluation. The choice for a DSST-based task was deliberate because it proved sensitive to detect cognitive complaints and changes in cognitive functioning in clinical samples[33, 34]. As this is a cognitive healthy sample, it is unsurprising that participants made little mistakes. Generally, cognitive performance can be viewed as a trade-off between accuracy and speed. Here, accuracy showed a ceiling effect (with reduced variability) while speed is a more sensitive measure. Only the number of trials showed a learning effect over time, with a slight increase of trials during the first half of the ESM period followed by a stabilization. Additionally, more trials were completed towards the end of the day.

The relationship between mood and the accuracy outcome reflected a positive interaction effect between positive affect, negative affect, and the percentage of correct trials. In situations were negative affect is high, participants also tend to make more mistakes, an effect that is strongest when positive affect is low. Zooming in on individual mood items, only cheerful and anxious seemed to be associated with the accuracy outcome. Therefore, it has merit to unpack the positive and negative mood aggregations to get relevant information and clues for clinical practice. A possible explanation could be that people are less able to focus on a task when they feel anxious. This negative influence of mood on cognitive performance is observed in clinically depressed patients and might be caused by distractions due to ruminations[35, 36]. Here, participants who got distracted during the task also made more mistakes. As distraction was assessed after task completion, it is possible that participants who noticed that they made mistakes, consequently scored higher on distraction. Overall, the explained variance for accuracy in relation to mood and contextual factors is neglible (3%) and combined with a ceiling effect it seems to be an irrelevant chance finding in a population without cognitive complaints.

A small positive association was found between mood (positive affect and more specifically cheerful) and the speed outcome. Participant’s who were more cheerful also completed more trials irrespective of learning effects. With regard to contextual and intrapersonal factors, a small negative association was found between age and speed, indicating that older participants overall completed less trials. The original Digit Symbol Substitution task is known to be sensitive in identifying age-related performance and processing speed often explains a large part of the variance in these studies[37]. Our modified digital version of the task was also age-sensitive. With regard to gender, males seemed to perform slower compared to females, an effect that disappeared in the final model. In the original Digit Substitution tests, men also seem to perform less well when averaged[38, 39]. In this convenience sample however, females were overrepresented (73%) and further research is needed.

Similar to the accuracy outcome, higher distraction was associated with fewer completed trials within a 30-second session. Here, the overall explained variance is clearly higher (36%). There is more variation over time with only a small learning effect. Indicating that the speed outcome is more suited to assess cognition in the current sample.

Several daily life factors were explored. Only distraction was associated with cognition, whereas other factors such as activity-related stress, company, and being able to focus were not. One other study looked at situational cues in relation to cognitive performance within an ESM paradigm. They found that working memory performance did not differ for people at work versus at home, but that short-term memory improved during worktime[40]. Possibly, processing speed is less sensitive to contextual changes.

Notably, fatigue did not vary significantly over time and had no effect on cognition. This was surprising, since other studies with a young population show a negative impact of tiredness on mental processing and increased difficulties with focusing on a task[41–43]. However, the mDSST was only 30 seconds while a standard cognitive assessment is longer (often 2 minutes). It is likely that the association of cognition with fatigue only occurs in longer or more demanding tasks, which are not suited to the ESM paradigm.

### Strengths and limitations

The PsyMate^™^ app with the mDSST can be used on an individual’s own smartphone and is not restricted to the provided iPod. The use of cognition tasks on smartphones is feasible[50, 51]. By using iPod devices across participants, the device specifications during the initial validity were standardized. In the early stages of task development, uncertainty about test characteristics, design choices, and device specifications exists. The use of the same device, the iPod, reduced the uncertainties about factors that might influence outcome across the study sample. In later stages, the influence of different devices (i.e., own smartphones) will become less problematic as the goal shifts towards an evaluation of within-person variability for clinical purposes.

Additionally, the mDSST was developed in an inter-professional context. Researchers (both in mental health and somatic care), physicians, neuropsychologists, clinicians, and software developers worked together to accomplish a tool that can prospectively be used across disciplines and in daily practice.

Although the study has several advantages, limitations need to be kept in mind. First, our sample was mainly restricted to female students (70% women, 61% students, median age was 23). The study, however, was intended as a pilot study using convenience sampling to assess initial feasibility and validity. The mDSST has shown merit for daily life assessment and age sensitivity of the mDSST could already be indicated. Nonetheless, using a more heterogeneous population, a broader age range (through stratification), as well as populations with cognitive impairments, will increase knowledge about task sensitivity as well as a more diverse examination of between- and within-person variance in task performance.

Second, technical problems have influenced the study outcomes. The beep questionnaire was only abailable for ten minutes. When participants initiated the questionnaire within the ten-minute boundary, the software should allow them to finish the task. However, the PsyMate^™^ app stopped after 10 minutes sharp, which resulted in 15 unfinished and interrupted tasks. The number of trials statistic was unreliable in these cases. Furthermore, the first participants indicated not hearing the beep sound (leading to eighteen missed beeps). This problem was resolved by a system update that enabled a louder and more intrusive beep sound. The technological issues concerning the mDSST seem unlikely to have influenced the performance outcome; the proportion of correct answers was high. Nevertheless, participants experienced those issues as unpleasant and in the future a more reliable technology should be used.

Finally, while reflecting on the task, two participants reported making mistakes by accidentally pressing the wrong symbol since the buttons were too small. In addition, sixteen participants reported that they made mistakes due to the slow processing of the iPod. The mDSST could be improved by using smartphones with a larger screen so that the size of the buttons is increased. Another option would be to rotate the screen into landscape mode.

### Future direction

In light of the current study results, several questions still remain. Valuable, but limited information on the psychometric properties of the 30-second mDDST is gathered. It would be interesting to examine if the time interval can be further decreased (e.g., to 15 seconds) and still yields reliable data. A shorter duration could increase the feasibility and decrease the influence of distractions. The outcome measures of the task can be extended to include response time (milliseconds) to get an idea about the influence of distractions on task performance. Future research should investigate construct validity by comparing performance to the paper-and-pencil version of the DSST. This study is in progress. Sleep quality was assessed using the morning questionnaire, but not taken into account here due to power problems. Poor sleep quality can negatively influence cognitive performance during the day[13]. More attention needs to be paid to the influence of sleep quality and fatigue on cognitive performance in daily life. Smartwatches exist that can accurately track sleep patterns. It would be interesting to link objectively gathered sleep data to ESM cognition and fatigue outcomes.

The mDSST predominantly focuses on processing speed, but other tasks measuring additional cognitive constructs could be designed for use in an experience-sampling paradigm. This would allow to compute the discriminant validity, as was done by Sliwinski and colleagues[21]. However, it is unclear whether a battery of mobile cognition tasks is necessary for clinical purposes. Insight into daily cognitive fluctuations may be possible with an aspecific cognition task. Repeated cognitive testing using ESM technology do not allow for a conclusive assessment across cognitive constructs, cross-sectional test batteries are more suited for this purpose. Gaining a general sense of cognitive functioning in relation to other domains can provide concrete ideas on how to deal with cognitive deficits that are individually relevant during everyday life. Although in this study, the various contextual factors did not show an effect on cognitive performance it still seems valuable to examine possible links more closely. All these factors arguably influence daily cognitive functioning and should further be explored in the context of the rehabilitation process.

### Clinical implications

This study is moving away from a classic cross-sectional assessment of cognition to an ecological assessment of cognitive variation. The combination of the mDSST with experience sampling allows for an examination of the link between cognition and contextual and intrapersonal information. ESM is used in clinical assessments and to implement in situ interventions in various populations. Using this method helps to raise awareness for variability patterns in everyday life and it is used to support self-management and improve well-being[15]. Thus, making ESM a valuable tool to supplement assessments of behavior and mood, with the monitoring of cognitive abilities and its daily fluctuations.

Cognitive impairments are known to influence recovery and self-care behavior in various populations. In schizophrenia and depression, there is evidence that cognitive deficits contribute to poor psychosocial functioning[44, 45], while in bipolar disorders there is an association between cognitive dysfunction and the course and length of the illness[46]. A study by Cameron et al. [2010] showed that in patients with heart failure, cognitive problems hindered decision-making[47]. Individuals with diabetes, who experienced greater cognitive difficulties, were less likely to remain adherent to exercise or diet[48]. Teaching individuals self-management techniques is generally recommended for rehabilitation purposes, for example after a stroke[49].

Understanding oneself and one’s (cognitive) abilities is important for self-management. By monitoring cognition with ESM and by examining the results afterwards, knowledge can be gained about previously nontransparent patterns between behavior, mood, and cognition, facilitating this understanding[15]. Learning when difficulties arise and under which circumstances, could help patients to adjust their tasks accordingly. Individuals might thus plan their days according to their cognitive abilities and, for example, schedule resting moments when cognitive exhaustion occurs. Keeping track of minor changes towards recovery motivates patients and helps clinicians to adapt treatment plans. Cognition tasks like the mDSST can be helpful in supporting future treatment, prevention, and rehabilitation.

## Conclusions

Adding a digital cognition task to an experience-sampling paradigm proved to be feasible in healthy individuals. The mDSST is promising and sensitive to detect cognitive variability in relation to mood, intrapersonal, and contextual factors. Although the task seems promising, further exploration is needed in more diverse age samples and in clinical populations with cognitive complaints. The implementation could be improved by providing some minor changes to the task (e.g., larger buttons or screen for visibility). It is clinically relevant to grasp how cognition fluctuates over time and relates to daily life functioning. By providing patients and clinicians with feedback on this data, cognitive rehabilitation and self-management can be improved.

## Supporting information

S1 Appendix Experience sampling items. (DOCX)

S1 Table Individual multilevel regression analyses. (DOCX)

S2 Table Correlations between mood items. (DOCX)

S3 Table Multilevel stepwise regression analyses. (DOCX)

S1 Dataset (XLS)
