# Supplementary material for: Measuring within-day cognitive performance using the experience sampling method: A pilot study in a healthy population
Source: PLoS One. 2019 Dec 12;14(12):e0226409. doi: 10.1371/journal.pone.0226409 (PMC6907820; doi:10.1371/journal.pone.0226409)
Supplement: S1 Appendix — (DOCX) [file pone.0226409.s001.docx]

**S1 Appendix. Experience sampling items**

Experience Sampling Protocol: Beep Questionnaire

|  | **Item** | **7-point Likert scale or categorical options** |
| --- | --- | --- |
| 1 | I feel cheerful | 1 = not at all 4 = moderate 7 = very much |
| 2 | I feel energetic | 1 = not at all 4 = moderate 7 = very much |
| 3 | I feel insecure | 1 = not at all 4 = moderate 7 = very much |
| 4 | I feel relaxed | 1 = not at all 4 = moderate 7 = very much |
| 5 | I feel down | 1 = not at all 4 = moderate 7 = very much |
| 6 | I feel irritated | 1 = not at all 4 = moderate 7 = very much |
| 7 | I feel satisfied | 1 = not at all 4 = moderate 7 = very much |
| 8 | I feel lonely | 1 = not at all 4 = moderate 7 = very much |
| 9 | I feel enthusiastic | 1 = not at all 4 = moderate 7 = very much |
| 10 | I feel anxious | 1 = not at all 4 = moderate 7 = very much |
| 11 | I feel guilty | 1 = not at all 4 = moderate 7 = very much |
| 12 | I’m worrying about things | 1 = not at all 4 = moderate 7 = very much |
| 13 | I generally feel well at the moment | 1 = not at all 4 = moderate 7 = very much |
| 14 | What am I doing | work, school/housekeeping/self-care/relaxing/sport/eating, drinking /traveling, on the road/having a conversation/something else/nothing |
| 15 | I can do this well | 1 = not at all 4 = moderate 7 = very much |
| 16 | This is difficult for me | 1 = not at all 4 = moderate 7 = very much |
| 17 | I would rather be doing something else | 1 = not at all 4 = moderate 7 = very much |
| 18 | I am focused | 1 = not at all 4 = moderate 7 = very much |
| 19 | Where am I | at home/at someone else’s home/work, school/public space/on the road/somewhere else |
| 20 | Who am I with | partner/family/housemates/friends/colleagues/acquaintances/strangers, others / nobody |
| 21a | Company: I like this company | 1 = not at all 4 = moderate 7 = very much |
| 22a | Company: I would rather be alone | 1 = not at all 4 = moderate 7 = very much |
| 21b | Alone: I like being alone | 1 = not at all 4 = moderate 7 = very much |
| 22b | Alone: I would rather be in company | 1 = not at all 4 = moderate 7 = very much |
| 23 | I don’t feel well | 1 = not at all 4 = moderate 7 = very much |
| 24 | I am tired | 1 = not at all 4 = moderate 7 = very much |
| 25 | Since the last beep I have used | alcohol/medication/coffee, caffeine/smoking, nicotine/cannabis/other drugs/nothing |
| 26 | mDSST instruction screen |  |
|  | mDSST | 30 seconds task duration |
| 27 | I got distracted during the task | 1 = not at all 4 = moderate 7 = very much |
| 28 | This beep disturbed me | 1 = not at all 4 = moderate 7 = very much |
| 29 | Thanks! |  |
