# Supplementary material for: Measuring within-day cognitive performance using the experience sampling method: A pilot study in a healthy population
Source: PLoS One. 2019 Dec 12;14(12):e0226409. doi: 10.1371/journal.pone.0226409 (PMC6907820; doi:10.1371/journal.pone.0226409)
Supplement: S1 Table — (DOCX) [file pone.0226409.s002.docx]

**S1 Table. Individual multilevel regression analyses**

**Table. Multilevel Regression Analyses of the Explored ESM Items Separate for Number of Trials and Percentage of Correct Trials**

|  | **Number of trials** | | | | | | **Percentage of correct trials** | | | | | | | |
| --- | --- | --- | --- | --- | --- | --- | --- | --- | --- | --- | --- | --- | --- | --- |
|  | **B** | **SE** | **p** | | **95% CI** | | **B** | | **SE** | | **p** | | **95% CI** | |
| **Positive Affect** | .08 | .04 | .04* | .005, | | .16 | .62 | .19 | | .001* | | .24, | | .99 |
| - **Cheerful** | .12 | .03 | <.001* | .06, | | .18 | .54 | .15 | | <.001* | | .24, | | .85 |
| - **Energetic** | .06 | .03 | .02* | .01, | | .12 | .14 | .14 | | .32 | | -.14, | | .42 |
| - **Relaxed** | .01 | .03 | .78 | -.05, | | .06 | .51 | .14 | | <.001* | | .23, | | .80 |
| - **Satisfied** | .02 | .03 | .51 | -.04, | | .08 | .53 | .16 | | .001* | | .22, | | .83 |
| - **Enthusiastic** | .02 | .03 | .53 | -.04, | | .07 | .13 | .14 | | .34 | | -.14, | | .39 |
| **Negative Affect** | -.03 | .06 | .58 | -.14, | | .08 | -1.41 | .28 | | .000* | | -1.96, | | -.86 |
| - **Down** | -.04 | .03 | .24 | -.10, | | .03 | -.40 | .17 | | .02* | | -.74, | | -.06 |
| - **Insecure** | -.01 | .03 | .80 | -.08, | | .06 | -.62 | .18 | | <.001* | | -.96, | | -.27 |
| - **Irritated** | -.004 | .03 | .89 | -.06, | | .05 | -.54 | .14 | | <.001* | | -.81, | | -.27 |
| - **Lonely** | .02 | .04 | .68 | -.06, | | .09 | -.56 | .19 | | .003* | | -.94, | | -.19 |
| - **Anxious** | -.005 | .05 | .92 | -.10, | | .09 | -.90 | .25 | | <.001* | | -1.39, | | -.41 |
| - **Guilty** | -.03 | .04 | .41 | -.12, | | .05 | -.65 | .21 | | .002* | | -1.07, | | -.23 |
| **Fatigue** | -.01 | .02 | .52 | -.06, | | .03 | -.06 | .11 | | .59 | | - .29, | | .16 |
| **Worrying** | .02 | .03 | .54 | -.04, | | .07 | -.26 | .14 | | .07 | | -.54, | | .02 |
| **Focused** | .01 | .03 | .70 | -.04, | | .06 | .10 | .13 | | .44 | | -.16, | | .36 |
| **Distracted** | -.17 | .02 | <.001* | -.21, | | -.13 | -.46 | .11 | | <.001* | | -.67, | | -.25 |
| **Act. stress** | .02 | .03 | .44 | -.03, | | .07 | -.11 | .14 | | .44 | | -.38, | | .17 |
| **Location** | -.20 | .06 | .002* | -.32, | | .07 | .26 | .34 | | .45 | | -.41, | | .93 |
| **Company** | -.13 | .07 | .05 | -.26, | | .001 | -.02 | .35 | | .95 | | -.72, | | .67 |
| **Coffee use** | -.08 | .09 | .37 | -.25, | | .09 | -.37 | .46 | | .42 | | -1.28, | | .53 |
| **Age^2^** | -.001 | .0001 | <.001* | -.001, | | -.0005 | -.0002 | .0003 | | .50 | | -.0007, | | .0004 |
| **Gender** | -1.09 | .44 | .01* | -1.95, | | -.22 | .04 | .72 | | .96 | | -1.38, | | 1.46 |
| **Time^$^** | .36 | .03 | <.001* | .30, | | .43 | -.26 | .19 | | .17 | | -.63, | | .11 |
| **Hour** | .03 | .01 | <.001* | .01, | | .04 | -.03 | .04 | | .36 | | -.11, | | .04 |
| **Hour^2^** | .001 | .0002 | .001* | .0003, | | .001 | -.001 | .001 | | .30 | | -.004, | | .001 |

*Note.* CI = Confidence Interval, Act. Stress = Activity-related Stress. Location = dummy variable of being at home versus somewhere else. Company = dummy variable of being alone versus with others. Coffee use = dummy variable of coffee use since the last beep versus no coffee use. Age^2^ = quadratic function of age. Time^$^ = log-transformed replication score. Hour = hours within a day. Hour^2^ = quadratic function of hour. **p* < .05.
