# Supplementary material for: Measuring within-day cognitive performance using the experience sampling method: A pilot study in a healthy population
Source: PLoS One. 2019 Dec 12;14(12):e0226409. doi: 10.1371/journal.pone.0226409 (PMC6907820; doi:10.1371/journal.pone.0226409)
Supplement: S2 Table — (DOCX) [file pone.0226409.s003.docx]

**S2 Table. Correlations between mood items**

**Table A. Pearson Correlates for Single Positive Affect Items**

| **Variables** | **1** | **2** | **3** | **4** | **5** |
| --- | --- | --- | --- | --- | --- |
| **Overall variance** |  |  |  |  |  |
| **1. Cheerful** | - |  |  |  |  |
| **2. Energetic** | .74* | - |  |  |  |
| **3. Relaxed** | .47* | .42* | - |  |  |
| **4. Satisfied** | .59* | .55* | .57* | - |  |
| **5. Enthusiastic** | .67* | .66* | .48* | .62* | - |
| **Within-subject variance** |  |  |  |  |  |
| **1. Cheerful** | - |  |  |  |  |
| **2. Energetic** | .63* | - |  |  |  |
| **3. Relaxed** | .29* | .24* | - |  |  |
| **4. Satisfied** | .43* | .39* | .41* | - |  |
| **5. Enthusiastic** | .50* | .50* | .30* | .43* | - |

*Note.* All p < .001.

**Table B. Pearson Correlates for Single Negative Affect Items**

| **Variables** | **1** | **2** | **3** | **4** | **5** | **6** |
| --- | --- | --- | --- | --- | --- | --- |
| **Overall variance** |  |  |  |  |  |  |
| **1. Down** | - |  |  |  |  |  |
| **2. Insecure** | .45* | - |  |  |  |  |
| **3. Irritated** | .44* | .27* | - |  |  |  |
| **4. Lonely** | .49* | .40* | .30* | - |  |  |
| **5. Anxious** | .45* | .54* | .32* | .46* | - |  |
| **6. Guilty** | .33* | .34* | .31* | .30* | .48* | - |
| **Within-subject variance** |  |  |  |  |  |  |
| **1. Down** | - |  |  |  |  |  |
| **2. Insecure** | .33* | - |  |  |  |  |
| **3. Irritated** | .40* | .18* | - |  |  |  |
| **4. Lonely** | .35* | .25* | .20* | - |  |  |
| **5. Anxious** | .31* | .37* | .22* | .28* | - |  |
| **6. Guilty** | .27* | .20* | .21* | .18* | .29* | - |

*Note.* All *p< .001.
