# Supplementary material for: Measuring within-day cognitive performance using the experience sampling method: A pilot study in a healthy population
Source: PLoS One. 2019 Dec 12;14(12):e0226409. doi: 10.1371/journal.pone.0226409 (PMC6907820; doi:10.1371/journal.pone.0226409)
Supplement: S3 Table — (DOCX) [file pone.0226409.s004.docx]

**S3 Table. Multilevel stepwise regression analyses**

**Table A. Multilevel Stepwise Forward and Backward Regression Analyses of Individual ESM Items for Number of Trials**

|  | **Number of trials** | | | | | | |
| --- | --- | --- | --- | --- | --- | --- | --- |
|  | **B** | | **SE** | **p** | **95% CI** | | |
| **Forward (overall)** |  |  | | < . 001* | |  |  |
| **• Cheerful** | .10 | .03 | | < . 001* | | .05, | .16 |
| **• Location** | -.003 | .06 | | .96 | | -.13, | .12 |
| **• Distraction** | -.19 | .02 | | < . 001* | | -.23, | -.15 |
| **• Time^$^** | .35 | .03 | | < . 001* | | .28, | .41 |
| **• Hour** | .13 | .05 | | .01 | | .03, | .23 |
| **• Hour^2^** | -.004 | .002 | | .02 | | -.007 | -.0006 |
| **• Age^2^** | -.0008 | .0001 | | < . 001* | | -.001 | -.0006 |
| **Backward (overall)** |  |  | | < . 001* | |  |  |
| **• Cheerful** | .13 | .03 | | < .001* | | .07, | .19 |
| **• Irritated** | .07 | .03 | | .01* | | .02, | .12 |
| **• Age^2^** | -.0008 | .0001 | | < .001* | | -.001, | -.0006 |
| **• Time^$^** | .35 | .03 | | < .001* | | .29, | .42 |
| **• Hour** | .12 | .05 | | .02* | | .02, | .22 |
| **• Hour^2^** | -.004 | .002 | | .03* | | -.009, | -.0004 |

*Note.* CI = Confidence Interval. Age^2^ = quadratic function of age. Time^$^ = log-transformed replication score. Hour = hours within a day. Hour^2^ = quadratic function of hour. **p* < .05.

**Table B. Multilevel Stepwise Forward and Backward Regression Analyses of Individual ESM Items for Percentage of Correct Trials**

|  | **Percentage of correct trials** | | | | | | |
| --- | --- | --- | --- | --- | --- | --- | --- |
|  | **B** | | **SE** | **p** | **95% CI** | | |
| **Forward (overall)** |  |  | | < . 001* | |  |  |
| **• Cheerful** | .33 | .17 | | .06 | | -.007, | .66 |
| **• Relaxed** | .21 | .16 | | .20 | | -.11, | .52 |
| **• Insecure** | -.36 | .18 | | .05 | | -.72, | .002 |
| **• Irritated** | -.27 | .15 | | .07 | | -.57, | .02 |
| **• Distracted** | -.43 | .11 | | < .001* | | -.63, | -.22 |
| **Backward (overall)** |  |  | | < . 001* | |  |  |
| **• Cheerful** | .36 | .17 | | .03* | | .04, | .69 |
| **• Relaxed** | .31 | .16 | | .05 | | .003, | .62 |
| **• Anxious** | -.69 | .26 | | .01* | | -1.19, | -.19 |
| **• Location** | .75 | .35 | | .04* | | .05, | 1.44 |
| **• Distracted** | -.53 | .11 | | < .001* | | -.74, | -.32 |

*Note.* CI = Confidence Interval. **p* < .05.
